# Early maternal relational traumatic experiences and psychopathological symptoms: a longitudinal study on mother-infant and father-infant interactions

**DOI:** 10.1038/srep13984

**Published:** 2015-09-10

**Authors:** Renata Tambelli, Silvia Cimino, Luca Cerniglia, Giulia Ballarotto

**Affiliations:** 1 Dipartimento di Psicologia Dinamica e Clinica Sapienza University of Rome, Piazzale Aldo Moro, 5, 00185 Roma, Italy; 2 Dipartimento di Psicologia Dinamica e Clinica Sapienza University of Rome, Piazzale Aldo Moro, 5, 00185 Roma, Italy; 3 Department of Psychology International Telematic University Uninettuno, Corso Vittorio Emanuele II, 39 00186 Roma - ITALIA; 4 Psicologia dinamica e clinica Department Sapienza University of Rome, Piazzale Aldo Moro, 5, 00185 Roma, Italy

## Abstract

Early maternal relational traumas and psychopathological risk can have an impact on mother-infant interactions. Research has suggested the study of fathers and of their psychological profiles as protection or risk factors. The aim of the paper is to assess the quality of parental interactions during feeding in families with mothers with early traumatic experiences. One hundred thirty-six (N = 136) families were recruited in gynecological clinics: Group A included families with mothers who experienced early sexual/physical abuse; Group B was composed of families with mothers who experienced early emotional abuse or neglect; and Group C comprised healthy controls. The subjects participated in a 10-month longitudinal protocol [at the fourth month of pregnancy (T_0_), 3 months after child birth (T_1_), and 6 months after child birth (T_2_)] that included an observation of mother-infant and father-infant interactions during feeding (Scala di Valutazione dell’Interazione Alimentare [SVIA]) and a self-reporting 90-item Symptom Checklist-Revised (SCL-90-R). Maternal higher rates of depression and early traumatic experiences of neglect and emotional abuse predicted more maladaptive scores on the affective state of the dyad SVIA subscale. Paternal anxiety predicted more severe levels of food refusal in the child during feeding.

Many authors have found that early relational traumatic experiences (RTEs) may predict the onset of psychopathological symptoms during an individual’s lifetime^1^,^2^ and that women who have suffered sexual/physical or emotional abuse and neglect can demonstrate anxiety, depression, and other severe psychological difficulties during their transition to motherhood^3^. Pregnancy can, in fact, trigger the woman’s attachment system and activate her own representations based on her relationship with her parents^4^. The weight of these traumatic experiences with respect to the onset of psychopathological problems in mothers is more powerful if the experience has been lived precociously; therefore, the abuse that occurred in the mothers’ first 5 years of life is more likely to foster depression, anxiety, post-traumatic stress disorder (PTSD)^5^, and sleep disturbances, as well as a loss of concentration and interest in activities once enjoyed^6^. In the affected mothers’ children, these maternal psychopathological symptoms can be associated with poor social and cognitive outcomes during infancy and toddlerhood^7^, impaired psychological functioning in adolescence, compromised empathy skills (which may be associated with problems in moral reasoning and behaviors), weak school performance, anxiety, inadequate self-regulatory capacities, and attachment insecurity^8^,^9^,^10^,^11^,^12^,^13^.

During the past 20 years, these considerations have given birth to several different branches of research and intervention methodologies: one of them addresses maternal psychological problems subsequent to abuse and/or maltreatment, both in the childhood history and in the current family situation (marital maltreatment)^14^,^15^,^16^,^17^. Another branch of research addresses PTSD and post-traumatic stress syndrome (PTSS) in mothers who are survivors of traumatic experiences, such as sexual/physical abuse, natural disasters, parental loss, wars, terrorism, and extreme traumatic situations such as the Holocaust^18^,^19^,^20^. Other authors, mostly using the attachment theory framework, have focused on the parenting characteristics of mothers who have experienced traumatic events and have linked their often diminished parenting to a lack of mentalization^21^, which in turn is related to their past experiences. It has been shown that mothers who have lived through traumatic experiences and developed PTSDs are more likely to display hostile/intrusive parenting behaviors^22^,^23^ over time (starting during their children’s infancy and continuing in their children’s toddlerhood) and have children with internalizing and externalizing problems^24^,^25^. To assess the quality of parent-infant interactions, most studies in this field have focused on play routines, whereas only a few have observed feeding interactions^26^. Nevertheless, many authors have suggested that feeding interactions between parents and their children may be considered important contexts in which children learn to identify and to give sense to verbal and non-verbal communications^27^,^28^. This intersubjective process is suggested to represent a basis for attachment quality and for emotional/behavioral functioning^29^. Brazelton and colleagues^30^ described the specific characteristics of an interaction between traumatized mothers and their infants. The authors demonstrated how these interactions are shaped by emotional unavailability, a maternal flat affect, and frequent gaze aversion from the infant during daily routines such as feeding or breast-feeding^31^,^32^,^33^. More recently, it has been suggested that the psychopathological risk subsequent to traumatic events can be intergenerationally transmitted to offspring, both behaviorally/emotionally and genetically^34^,^35^. The latter hypothesis stems from recent epigenetic theories that emphasize the interaction between genes and environment^36^,^37^,^38^. Notably, neurobiological studies on trauma^39^ to date have mainly considered severe traumas (e.g., sexual violence, wars) and their common association with PTSD^1^. Yet, other studies have addressed more hidden—and just as problematic—types of trauma related to emotional abuse or neglect that can alter the amygdala circuit, that is considered to support maternal responsiveness and attunement with the child^40^,^41^,^42^,^43^,^44^. In recent years, the fathers’ role has also been addressed as a protective and adjunct risk factor for the onset of psychological difficulties in children^45^,^46^, due to changes in the families’ organization, which nowadays includes shared responsibilities between mothers and fathers in the rearing of children (e.g., in feeding and sleeping routines), with fathers involved in the care of their sons and daughters as much as mothers, who are now more often employed outside the home^9^. Lamb^47^ suggested that fathers interact with their children in a specific and unique fashion that is rather distinct from the way in which mothers interact with their children; fathers’ relational patterns with their children, in fact, seem more often characterized by physical contact and rough-and-tumble play, and this issue seems to have a specific role in supporting the child’s emotional-regulation processes^48^,^49^. For these reasons, it appears important to consider fathers in those studies which intend to assess the quality of parent-infant interactions, and although a growing number of scientific papers are considering the fathers’ role in children’s developmental and psychological outcomes, literature describing the use of observational tools with daily routines (e.g., play and/or feeding) remains scarce^50^.

Similarly, there is a dearth of research on non-clinical populations (i.e., subjects without any psychiatric diagnosis, recruited either from health-related services or from the general population), whereas the relationships among parental psychopathological risks, their traumatic experiences, and children’s psychological functioning have been widely and longitudinally studied in clinical samples with parents and children typically diagnosed with PTSD or PTSS in comorbidity with postnatal depression (PND) or clinical depression^51^.

Bearing the above literature in mind, we intended to study the quality of mother-infant and father-infant interactions in families with mothers who have experienced relational traumas, such as sexual/physical or emotional abuse and neglect (as defined by D’Andera and colleagues^52^), but who did not develop PTSDs or other psychiatric problems, considering parental psychopathological risks.

## Objectives and Methods

The research described here was approved by the Ethical Committee of the Psychology Faculty at Sapienza, University of Rome, before the start of the study and in accordance with the Declaration of Helsinki. Written informed consent was obtained from each of the study participants.

The general aim of the study was to longitudinally assess the quality of mother-infant and father-infant interactions during a daily routine, such as feeding interactions with children at ages 3 and 6 months, in families with mothers who have experienced early RTEs, such as physical/sexual abuse and emotional abuse/neglect, in the first 5 years of life. To this end, we subdivided our sample into three groups: families with mothers who have experienced physical/sexual abuse (Group A); families with mothers who have experienced emotional abuse/neglect (Group B); and families with mothers who have had no traumatic experiences (Group C). Our hypothesis was that even those mothers who have faced early RTEs but did not suffer from psychiatric disorders in their life histories could be at risk for the onset of psychopathological symptoms after their children’s births, as well as difficulties in their interactions with their children. We also hypothesized that early maternal RTEs could affect the quality of both the mothers’ and the fathers’ interactions with their children in association with specific paternal psychological profiles (e.g., fathers with anxiety/depression or obsessive-compulsive symptoms, as suggested by Cimino and colleagues^53^); we additionally assumed that different maternal relational traumas could be associated with different interactional difficulties between parents and their children.

The specific objectives of this study were:

- (a) to longitudinally assess the quality of relational mother-infant and father-infant interactions in the three groups during meals;
- (b) to longitudinally assess the severity of the psychopathological risks of mothers and fathers in the three groups;
- (c) and to verify whether maternal early trauma affected the quality of parent-child interactions during feeding while considering the mothers and fathers’ psychopathological risks.

### Subjects and procedure

Over a 5-year period, 897 families in north-central Italy addressed a group of gynecologic services during women’s pregnancies. In accordance with the gynecologists and following a program of prevention of maladaptive outcomes in children of parents with psychopathological risks, we administered a research protocol to both mothers and fathers starting at the fourth month of pregnancy (T_0_). Administered were the following: 1) a 90-item Symptom Checklist-Revised (SCL-90-R^54^); and 2) an anamnestic questionnaire addressing past or current potentially traumatic experiences.

For the present study, we excluded families in which mothers faced the sorts of traumatic experiences not addressed in this study, such as natural disasters, terrorist attacks, earthquakes, and the like (N = 11); we considered only those mothers having experienced physical/sexual abuse and emotional abuse/neglect (N = 150) and families in which women did not have traumatic experiences (N = 736). We also excluded those families who had other children (N = 353) and all parents who exceeded the clinical cut-offs of SCL-90-R for the Italian population (N = 168)^55^ or who had been diagnosed with psychiatric disorders in the past (according to DSM-IV criteria^56^; N = 35). We also excluded from the present study those families in which the pregnancy was interrupted due to various difficulties or in which the fetus had medical problems or malformations (N = 83). At T_1_, after the delivery, we excluded families if the mother and father were not personally handling the child’s care and nutrition (N = 63). Moreover, the N = 48 families did not agree to continue as participants in the study. In the remaining sample group (N = 136), no father reported potentially traumatic experiences, and the subjects formed the following three groups: Group A (N = 39; families with mothers who have experienced physical/sexual abuse); Group B (N = 42; families with mothers who have experienced emotional abuse/neglect); and Group C (N = 55; families with mothers who have had no traumatic experiences). At T_1_ (at the children’s age of 3 months), we administered (to both mothers and fathers, who completed the questionnaires independently) the SCL-90-R and the SVIA (Scala di Valutazione dell’Interazione Alimentare—a scale with which to evaluate parent-child interaction during feeding, which is an Italian adaptation^57^ of the Feeding Scale^58^). All parent-child interactions were observed and recorded (20-minute videos) at their homes during lunch at midday; the feeding interactions, which occurred during a part of one regular meal, were observed separately: the mother-child interactions on one day, and the father-child interactions on another. The videos were recorded by psychologists specifically trained in the use of this observational tool and were coded by two trained independent raters who watched the videos and scored them on the basis of the manual^57^ while using both a paper-pencil system and a coding software program designed for the computation of scores on each subscale. We also administered the same anamnestic questionnaire we used at T_0_ to check whether parents had encountered traumatic experiences between T_0_ and T_1_. No parent reported having traumatic experiences during that period of time. At T_2_ (at the children’s age of 6 months) we repeated the protocol used at T_1_. Table 1 shows the demographic characteristics of participants by group.

The possible occurrence of traumatic experiences in the parents’ lives was controlled and, also at T_2_ (as at T_1_), no potentially traumatic experience was reported by mothers or fathers. All the families (N = 136) participated in the study at T_2_.

Most of the families recruited for the study (89%) had a middle socio-economic status, and a large majority (89%) comprised intact family groups. Ninety-three percent of the families were Caucasian, and 75% relied on more than one income. All the babies were breast- and formula-fed (mixed-fed), and all the fathers took part in the children’s caretaking and feeding routines.

### Measures

At T_0_, T_1_, and T_2_, all parents were administered the SCL-90-R^54^ independently. Also, mother-infant and father-infant nutrition interactions were video-recorded at T_1_ and T_2_, and evaluated via the SVIA^57^.

#### SCL-90-R

The SCL-90-R, a self-report questionnaire that gives a standardized measure of the current psychological and/or psychopathological status of a subject, can be applied in non-clinical or psychiatric adult and adolescent populations. It provides a wide range of information on the current subjective experience of psychological well-being and distress, and serves as a screening tool in both clinical and research settings. The scores obtained are interpreted based on nine primary dimensions: 1) somatization; 2) obsessive-compulsive behavior; 3) interpersonal sensitivity; 4) depression; 5) anxiety; 6) hostility; 7) phobic anxiety; 8) paranoid ideation; and 9) psychoticism. It includes a Global Severity Index (GSI) that is used to determine the severity and degree of psychological distress with respect to the nine primary dimensions measured. Prunas and collaborators demonstrated satisfactory internal consistency of the Italian version of the SCL-90-R in adolescents and adults (alpha coefficient, 0.70–0.96), with a clinical cut-off score indicating psychopathological risk^55^.

The SCL-90-R has been widely used to assess various psychopathological symptoms, such as depressive symptoms in mothers and fathers^59^.

#### SVIA

The SVIA is the Italian adaptation of the Feeding Scale^58^, which can be applied to children that are 0–36 months old. It measures interactive behaviors and identifies normal and/or risky relational modes between a parent and child during feeding exchanges^57^. Parent-infant interactions during feeding are recorded for at least 20 minutes, after which a wide range of interactive parent-infant behaviors are coded and evaluated.

The SVIA consists of 41 items distributed among four subscales: 1) parents’ affective states (index of the parents’ affective states); 2) interactive conflict (index of interactions characterized by conflictual, non-collaborative, and non-empathetic communication); 3) food-refusal behavior (habits associated with challenged status regulation during meals and with limited food consumption); and 4) affective state of the dyad (index of the extent to which the infant’s feeding patterns are, or are not, the result of an interactive regulation to which both partners contribute). The scores, measured on a 4-point Likert scale ranging from 0 to 3 (none, a little, quite a bit, a lot) for each subscale, were compared with standard values from the Italian standardized sample.

Inter-evaluator agreement for SVIA items is generally good to excellent (Pearson r values = 0.7–1.0 for a group of 182 control infants and 0.9–1.0 for a group of 182 infants with nutritional disorders). The instrument shows good reliability in terms of internal consistency (Cronbach’s alpha = 0.79–0.96).

### Data analysis

Before performing the analyses, the variables’ normality was preliminarily ascertained. All the variables were normally distributed; correlational analyses showed that, in all groups, the relations between mothers’ and fathers’ SCL-90-R dimensions were not significantly, or even slightly, related (<0.30); the internal consistency was adequate for all variables and higher than 0.80.

In the present study, parents’ SCL-90/R baseline scores were assessed at T_0,_ with the aim of having an assessment of the parents’ psychopathological risks before their children’s births. These scores served as exclusion criteria (as indicated in the “Subjects and procedure” section, all parents who exceeded the clinical cut-offs of SCL-90-R for the Italian population [N = 168]). Therefore, data referring to T_0_ were not included in the statistical analyses. Groups A, B, and C were compared through analyses of multivariate variance (MANOVAs). The time elapsed between the two sessions (from T_1_ to T_2_) was treated as a within-subject factor, and belonging to a research group was treated as a between-subjects factor. Bonferroni’s post hoc tests were applied. The calculated *p* values are reported with their respective F statistics and degrees of freedom (df), with values <0.05 being accepted as significant. Mean values are reported with standard deviations (SDs). Finally, two hierarchical regression analyses were conducted to investigate the influence of specific types of trauma (physical/sexual abuse or emotional abuse/neglect) and of SCL-90-R subscales on the relational dimensions of the Feeding Scale in mother-infant and father-infant interactions. In all the analyses we conducted, the child’s gender showed no significant effect on the variables. All analyses were performed with SPSS software (Version 18.0).

## Results

### Longitudinal assessment of the quality of mother-infant and father-infant interactions by group

A series of MANOVAs on SVIA subscale scores for mothers in Groups A, B, and C at T_1_ and T_2_ showed main effects of the groups (*p* < 0.001) with no time-point effect and no interaction effect. Bonferroni’s post hoc tests demonstrated that the mothers in Group B had significantly higher (i.e., more maladaptive) scores, both at T_1_ and T_2_, than Group A on the mother’s affective state (*F*_1,76_ = 21.81; *p* < 0.001) and interactive conflict (*F*_1,76_ = 68.75; *p* < 0.001) subscales. The scores of the mothers in Group C on all SVIA subscales were significantly lower (i.e., more adaptive; *p* < 0.001) than Groups A and B, both at T_1_ and T_2_. The mothers’ average scores and exact *p* values (for significant differences) for each SVIA subscale, at T_1_ and T_2_, are reported in Table 2.

A series of MANOVAs on SVIA subscale scores for the fathers of Groups A, B, and C revealed main effects of the groups (all *p* < 0.001) with no time-point effect and no interaction effect. Similar to our findings with regard to the mothers, Bonferroni’s post hoc tests demonstrated that the fathers in Group B had significantly higher (i.e., more maladaptive) scores both at T_1_ and T_2_ than Group A on the child food refusal (*F*_1,76_ = 199.724; *p* < 0.001) subscale. The scores of the fathers in Group C on all SVIA subscales were significantly lower (i.e., more adaptive; *p* < 0.05) than those of Groups A and B, both at T_1_ and T_2_. The fathers’ average scores and exact *p* values (for significant differences) for each SVIA subscale, at T_1_ and T_2_, are reported in Table 3.

For the present study, the inter-rater agreement between the two coders (specifically trained psychologists who were blind to group status) was good (Pearson r values = 0.74–0.89).

### Longitudinal evaluation of mothers’ and fathers’ psychopathological risk profiles by group

A series of MANOVAs on SCL-90-R mothers’ subscale scores showed a main effect of the group (*p* < 0.001) with no time-point effect and no interaction effect between time point and group. Bonferroni’s post hoc tests demonstrated that the mothers in Group B had significantly higher scores at T_2_ than Group A on the somatization (F_1,76_ = 7.91; *p* < 0.001), depression (F_1,76_ = 8.56; *p* < 0.01), and paranoid ideation (F_1,76_ = 8.96; *p* < 0.001) subscales. The mothers’ SCL-90-R scores on somatization, depression, and paranoid ideation exceeded the clinical cut-offs for the Italian population^55^. The scores of the mothers in Group C on all SCL-90-R subscales were significantly lower (i.e., more adaptive; p < 0.01) than those of Groups A and B, both at T_1_ and T_2_. The mothers’ average scores and exact *p* values (for significant differences) for each SCL-90-R subscale at T_2_ are reported in Table 4. Data regarding T_1_ are not shown in Table 4 as there are no significant differences in scores between the two points of assessment.

With regard to the fathers’ SCL-90-R evaluations, similar to that of the mothers, a series of MANOVAs on the SCL-90-R fathers’ subscale scores showed a main effect of the group (*p* < 0.001) with no time-point effect and no interaction effect between time point and group. Bonferroni’s post hoc tests demonstrated that the fathers in Group B had significantly higher scores, both at T_1_ and T_2_, than Group A on the obsessive-compulsive (F_1,76_ = 6.94; *p* < .001), anxiety (F_1,76_ = 9.78; *p* < 0.01), and hostility (F_1,76_ = 4.18; *p* < 01) subscales. The fathers’ scores in Group C on all SCL-90-R subscales were significantly lower (*p* < 0.01) than those in Groups A and B, both at T_1_ and T_2_. The fathers’ average scores for each SCL-90-R subscale at T_2_ are reported in Table 5. Data regarding T_1_ are not shown in Table 5 as there are no significant differences in scores between the two points of assessment.

No fathers’ scores exceeded the cut-offs for the Italian population^55^.

### Quality of parent–child interactions during feeding considering maternal early trauma and mothers’ and fathers’ psychopathological risks

Two regression analyses were conducted separately for mothers and fathers to investigate the influence of different types of maternal trauma and of all nine SCL-90-R subscales on all four of the relational dimensions of the SVIA in mother-infant and father-infant interactions at T_2_. The results showed that only when higher maternal scores on depression interact with early traumatic experiences of neglect and emotional abuse did they predict higher (and more maladaptive) scores on the affective state of the dyad SVIA subscale (*p* < 0.01). Further, early maternal traumatic experiences of sexual/physical abuse predicted higher scores on the mothers’ SVIA affective state subscale only when they interacted with the mothers’ higher somatization scores (*p* < 0.05). Moreover, early traumatic experiences, both sexual/physical and emotionally traumatic, in the mothers predicted higher (and more maladaptive) scores on paternal interactive conflict with the child during feeding (*p* < 0.05). With regard to the fathers, data analyses showed that paternal anxiety predicted more severe food refusal in the child during feeding (*p* < 0.05). No association or prediction was found between fathers’ psychopathological risks and maternal scores on the SVIA subscales.

The results and values of the regression analyses are shown in Table 6.

## Discussion and Conclusions

This study aimed to longitudinally assess the quality of mother-infant and father-infant interactions during the feeding of children at 3 and 6 months of age in families with mothers with early RTEs in a non-referred sample. Our hypothesis was that mothers who had early RTEs without being diagnosed with PTSD or other psychiatric disorders could be at risk for the onset of psychopathological (e.g., depressive) symptoms after their children’s births and could encounter difficulties in their interactions with their children. In addition, we intended to ascertain whether maternal RTEs could affect the quality of both the mothers’ and the fathers’ interactions with their children.

To our knowledge, this is the first study of early maternal relational trauma that has assessed the quality of parental interactions with their children while using an observational method to focus on a non-clinical population. Our results showed that mothers with early traumatic experiences (Groups A and B) had significantly more maladaptive interactions during the feeding of their children, both at 3 months and 6 months of age, when compared to mothers who had not experienced traumas (Group C). Specifically, mothers who had experienced emotional abuse or neglect (Group B) showed scores on the affective state and interactive conflict subscales that were higher than those of mothers who had experienced sexual/physical abuse (Group A), indicating that dyadic exchanges were more often characterized by feelings of sadness in the mother and by un-attuned, non-contingent interactions with her child. It is important to underline that mothers in both Groups A and B showed maladaptive interactions with their children that exceeded the clinical cut-offs for the Italian population^57^. This result is coherent with Kim, Trickett, and Putnam’s studies^60^ that demonstrated how the maternal experiences of emotional abuse and neglect can severely affect the quality of their interactions with their children during play or feeding. Our data indicate that in a non-referred sample (i.e., a group of non-psychiatrically diagnosed mothers), these forms of early traumatic experiences, as much as early sexual/physical abuse, can affect relations with the child. This result necessitates further attention. In fact, previous studies have shown mixed results: Some authors have suggested that sexual abuse can have a more severe impact on subjects’ psychopathology and has more frequently been associated with psychiatric diagnoses (e.g., PTSD), whereas other research has demonstrated that female victims of emotional abuse have a higher risk of psychopathological (including depressive) symptoms compared with women who have been exposed to sexual or physical abuse^61^. We speculate that early parental emotional abuse and neglect have an impact more severe than other forms of abuse on mother-infants interactions because they can specifically affect mothers’ perceptions of their competency in offspring-rearing. This poor perception of their competency as caregivers could be particularly active during feeding^62^. The same consideration can apply to our results regarding father-infant interactions. In our study, fathers in families with mothers who have experienced emotional abuse or neglect showed lesser-quality interactions with their children than fathers who were partners to women with sexual/physical abuse. Particularly, their exchanges were characterized by a child’s food refusal, both at T_1_ and T_2_. This result may be interpreted while considering the studies of Haycraft and Blissett^63^, who suggest that fathers are more likely than mothers to control the feeding and impose more pressure to the child in the attempt to make him/her eat; in doing so, they may provoke food-refusal behaviors in their children. Psychopathological risks in mothers and fathers appeared significantly more problematic for subjects belonging to Group B (families with mothers who have experienced emotional abuse and neglect) when compared to other groups. Mothers in this group exceeded the clinical cut-off for Italian population in the somatization, depression, and paranoid ideation subscales, whereas the fathers in Group B showed higher scores than the fathers in Groups A and C on the obsessive-compulsive, anxiety, and hostility subscales (but they did not exceed the clinical cut-offs for the Italian population). This result was valid with respect to children at both 3 months and 6 months of age, suggesting that while mothers tend to show symptoms belonging to the depressed/withdrawn/covert framework, fathers are more likely to exhibit overt psychology difficulties. This finding is coherent with those of several other studies^53^,^64^. We are impressed by the very peculiar configuration shown by families in Group B, in which mothers and fathers had more problematic dyadic interactions with their children, as well as higher levels of individual psychopathological risk. These data seem to suggest that the weight of early maternal relational trauma (both sexual/physical abuse, as most research indicates, and emotional abuse and neglect) is such that it strongly affects the whole familial functioning, influencing both individual and relational characteristics of family members. Moreover, the effect of early maternal relational traumas was stable over time, impeding the reversion of psychopathological symptoms that mothers often develop during pregnancy and after delivery, but which usually decrease after the first months postpartum^65^. Regression analyses showed that early maternal traumatic experiences, both sexual/physical and emotionally traumatic, and neglect in the mothers predicted more maladaptive scores on paternal interactive conflict with the child during feeding. We make the hypothesis, coherent with family and ecological theories^66^,^67^ and with previous studies^68^, that mother-infant and father-infant dyads are interconnected. However, no association or prediction was found between fathers’ psychological characteristics (i.e., psychopathological risks) and maternal scores on the SVIA subscales.

We also found that higher maternal scores on depression, in association with early traumatic experiences of neglect and emotional abuse, predicted mothers’ more maladaptive scores on the SVIA affective state of the dyad subscale. Moreover, early maternal traumatic experiences of sexual or physical abuse in association with mothers’ higher somatization scores predicted higher maternal scores on the mothers’ SVIA affective state of the mother subscale. These results are consistent with the studies of Cimino and colleagues^9^ that underlined the importance of maternal psychological/emotional functioning for the quality of early parent-infant interactions. With regard to fathers, coherent with Ramchandani^69^, data analyses showed that paternal anxiety predicted more severe food refusal in the child during feeding.

The present study has several strengths. We assessed maternal psychological functioning prior to pregnancy (at T_0_), which provided a baseline for mothers psychological characteristics that we were able to longitudinally compare with their subsequent scores on the same questionnaires (at T_1_ and T_2_). Moreover, we evaluated the links between specific types of early maternal traumas (sexual/physical and emotional abuse and neglect) and definite psychopathological parental symptoms while considering the impact of maternal traumatic experiences and of their psychopathological symptoms on the quality of both mothers’ and fathers’ interactions with their children. Further, we used an observational measure, which was specifically built for scoring both emotional and behavioral patterns, both individual (characteristics of the child and of the mother individually) and dyadic, to assess the quality of parent-infant interactions during feeding. We also considered fathers in our study, as recent literature recommends. Finally we focused on a non-clinical population instead of assessing psychiatrically diagnosed or multi-risk samples, as most research has done, which can be important for planning prevention/intervention programs, and also because women who have suffered traumatic experiences are reported to be significantly more likely to visit health-care providers prior to and during pregnancy, as well as postpartum^70^. This suggests that these periods can be a critical opportunity for intervention in order to interrupt the possible cycle of trauma^71^,^72^.

This study had some limitations. We used self-report—although well validated and widely used—questionnaires for the assessment of parental psychopathological risks. We also did not assess children’s emotional/behavioral functioning and temperamental characteristics, which might have a weight on parental interactional styles and on psychological functioning^73^. Parent-infant attachment was not considered, being instead a key issue for understanding and evaluating mother-infant and father-infant interactions, as well as for predicting future children’s emotional/cognitive and behavioral development. The present study did not focus on family support, in terms of help given to parents in the rearing of the child by grandparents and other relatives, who may also economically support the parents or moderate the effect of maternal or paternal psychopathology on their interactions with the child, as Steel has suggested^74^. Aside from the procedure to distinguish one form of trauma (e.g., sexual abuse) from other types of traumatic experiences (e.g., emotional abuse), which can overlap^3^, must be revised, as in this study we used only self-report measures, whereas it could be useful to include other tools, such as clinical interviews or report-form questionnaires completed by clinicians. Finally, the homogeneity of the sample in terms of cultural, geographical, and SES, limits the replication of this study in other countries or cultures.

## Additional Information

**How to cite this article**: Renata, T. *et al.* Early maternal relational traumatic experiences and psychopathological symptoms: a longitudinal study on mother-infant and father-infant interactions. *Sci. Rep.* **5**, 13984; doi: 10.1038/srep13984 (2015).

## Figures and Tables

**Table 1:** Demographic characteristics of participants by group.

Group	N	Sex of the child	Mean age ± SD			
			Mothers (years)	Fathers (years)	Infants at T1 (months)	Infants at T2 (months)
A	39	18 M, 21 F	32.8 ± 2.2	36.4 ± 2.1	3.2 ± 0.2	6.3 ± 0.6
B	42	20 M, 22 F	33.4 ± 2.5	36.1 ± 1.7	3.3 ± 0.3	6.5 ± 1.0
C	55	23 M, 22 F	31.4 ± 2.2	39.2 ± 2.4	3.1 ± 0.4	6.2 ± 0.6

**Table 2:** Mean SVIA subscale scores ± SD and p values at T1 and T2 by group for mothers.

	Group	A	B	C**	*P*
T1	Mother’s affective state	16.17 ± 2.28^a^	21.71 ± 1.49^b^*	10.22 ± 1.21^c^	*0.0008
	Interactive conflict	9.67 ± 1.33^a^	19.32 ± 1.80^b^*	4.31 ± 1.11^c^	*0.0004
	Food refusal	7.01 ± 1.11^a^	7.51 ± 1.82^a^	3.84 ± 1.22^b^	**
	Dyad affective state	6.27 ± 1.71^a^	6.93 ± 1.63^a^	3.74 ± 0.52^b^	**
T2	Mother’s affective state	15.13 ± 1.86^a^	20.91 ± 2.32^b^*	10.72 ± 1.45^b^	*0.0007
	Interactive conflict	9.57 ± 1.65^a^	18.71 ± 2.25^b^*	4.12 ± 1.33^c^	*0.0006
	Food refusal	7.19 ± 1.19^a^	7.20 ± 1.31^a^	4.17 ± 1.09^b^	**
	Dyad affective state	6.89 ± 0.78^a^	6.54 ± 1.46^a^	3.89 ± 0.32^b^	**

^*^Significantly higher scores.

^**^Group C has significantly lower scores than Group A and B on all SVIA sub-scales both at T1 and T2 (*p* < 0.001).

**Table 3:** Mean SVIA subscale scores ± SD and p values at T1 and T2 by group for fathers.

	Group	A	B	C**	*P*
T1	Father’s affective state	13.46 ± 2.36^a^	13.14 ± 2.59^a^	11.31 ± 1.85^b^	**
	Interactive conflict	9.78 ± 2.61^a^	9.67 ± 2.30^a^	4.42 ± 1.41^b^	**
	Food refusal	6.12 ± 1.46^a^	12.13 ± 1.21^b^*	3.08 ± 1.27^c^	*0.0007
	Dyad affective state	7.82 ± 1.96^a^	7.97 ± 1.61^a^	3.88 ± 0.69^b^	**
T2	Father’s affective state	13.39 ± 2.30^a^	13.11 ± 2.12^a^	11.11 ± 1.80^b^	**
	Interactive conflict	9.27 ± 1.60^a^	9.12 ± 2.31^a^	4.75 ± 1.55^b^	**
	Food refusal	6.38 ± 1.48^a^	12.43 ± 1.44^b^	3.51 ± 1.38^c^	*0.0008
	Dyad affective state	7.14 ± 0.62^a^	7.14 ± 0.62^a^	4.03 ± 0.45^b^	**

^*^Significantly higher scores.

^**^Group C has significantly lower scores than Group A and B on all SVIA sub-scales both at T1 and T2 (*p* < 0.001).

**Table 4:** Mean mothers’ SCL-90-R scores ± SD and p values by group at T2.

Mean ± SD	Group	A	B	C**	*p*
	SOM	0.92 ± 0.1^a^	1.06 ± 0.3^b^*	0.21 ± 0.4^c^	*0.0007*
	O-C	0.73 ± 0.2^a^	0.82 ± 0.5^a^	0.42 ± 0.3^b^	**
	I-S	0.86 ± 0.2^a^	0.74 ± 0.3^a^	0.32 ± 0.2^c^	**
	DEP	0.75 ± 0.3^a^	1.04 ± 0.3^b^*	0.53 ± 0.4^c^	*0.008*
	ANX	0.66 ± 0.5^a^	0.78 ± 0.6^a^	0.51 ± 0.5^b^	**
	HOS	0.78 ± 0.5^a^	0.75 ± 0.2^a^	0.44 ± 0.3^b^	**
	PHOB	0.84 ± 0.6^a^	0.95 ± 0.4^a^	0.61 ± 0.2^b^	**
	PAR	0.81 ± 0.2^a^	1.06 ± 0.6^b^*	0.71 ± 0.8^c^	*0.007*
	PSY	0.73 ± 0.9^a^	0.37 ± 0.4^a^	0.33 ± 0.2^b^	**

Cut-off for psychopathological risk in Italian population is ≥1 for men and women 55.

SOM: Somatization; O-C: Obsessive-Compulsive; I-S: Interpersonal Sensitivity; DEP: Depression; ANX: Anxiety; HOS: Hostility; PHOB: Phobic Anxiety; PAR: Paranoid Ideation; PSY: Psychoticism.

^*^Significantly higher scores.

^**^Group C has significantly lower scores than Group A and B on all SCL-90-R sub-scales both at T2 (*p* < 0.001).

**Table 5:** Mean fathers’ SCL-90-R scores ± SD by group at T2.

Mean ± SD	Group	A	B	C	*p*
	SOM	0.82 ± 0.1^a^	0.96 ± 0.2^a^	0.18 ± 0.3^b^	**
	O-C	0.74 ± 0.2^a^	0.90 ± 0.4 ^b^*	0.32 ± 0.4^c^	*0.0007*
	I-S	0.86 ± 0.2^a^	0.74 ± 0.5^a^	0.22 ± 0.3^b^	**
	DEP	0.75 ± 0.3^a^	0.84 ± 0.2^a^	0.33 ± 0.5^b^	**
	ANX	0.66 ± 0.5^a^	0.88 ± 0.6 ^b^*	0.42 ± 0.6^c^	*0.005*
	HOS	0.74 ± 0.5^a^	0.97 ± 0.2 ^b^*	0.34 ± 0.3^c^	*0.006*
	PHOB	0.74 ± 0.8^a^	0.75 ± 0.7^a^	0.51 ± 0.4^b^	**
	PAR	0.61 ± 0.3^a^	0.66 ± 0.5^a^	0.25 ± 0.5^b^	**
	PSY	0.72 ± 0.5^a^	0.74 ± 0.3^a^	0.23 ± 0.5^b^	**

Cut-off for psychopathological risk in Italian population is ≥1 for men and women 55.

SOM: Somatization; O-C: Obsessive-Compulsive; I-S: Interpersonal Sensitivity; DEP: Depression; ANX: Anxiety; HOS: Hostility; PHOB: Phobic Anxiety; PAR: Paranoid Ideation; PSY: Psychoticism.

^*^Significantly higher scores.

^**^Group C has significantly lower scores than Group A and B on all SCL-90-R sub-scales at T2 (*p* < 0.001).

**Table 6:** Results and Values of the Regression Analyses at T2.

SCL-90-R/Maternal Early Traumatic Experiences	SVIA			
Mother	R^2^	ß	t	p
*Depression•Neglect/Emotional Abuse*	*Affective State of the Dyad (mother)*			
	0.131	0.356	3.345	0.003**
*Somatization*•*Sexual/Phisical Abuse*	*Affective State of the Mother*			
	0.073	0.254	2.346	0.031*
*Neglect/Emotional Abuse and Sexual/Phisical Abuse*	*Affective State of the Fathers*			
	0.126	0.323	3.074	0.021*
**Father**	**R^2^**	**ß**	**t**	**p**
*Anxiety*	*Child’s Food Refusal*			
	0.143	0.321	3.151	0.014*

N.B. The subscales that are not shown in the Table are not statistically significant.

*p < 0.05; **p < 0.01.

^•^Association with.
